# Dobrava-Belgrade Hantavirus from Germany Shows Receptor Usage and Innate Immunity Induction Consistent with the Pathogenicity of the Virus in Humans

**DOI:** 10.1371/journal.pone.0035587

**Published:** 2012-04-24

**Authors:** Elena Popugaeva, Peter T. Witkowski, Mathias Schlegel, Rainer G. Ulrich, Brita Auste, Andreas Rang, Detlev H. Krüger, Boris Klempa

**Affiliations:** 1 Institute of Virology, Helmut-Ruska-Haus, Charité Medical School, Berlin, Germany; 2 Friedrich-Loeffler-Institut, Institute for Novel and Emerging Infectious Diseases, Greifswald-Insel Riems, Germany; 3 Institute of Virology, Slovak Academy of Sciences, Bratislava, Slovakia; Kantonal Hospital St. Gallen, Switzerland

## Abstract

**Background:**

Dobrava-Belgrade virus (DOBV) is a European hantavirus causing hemorrhagic fever with renal syndrome (HFRS) in humans with fatality rates of up to 12%. DOBV-associated clinical cases typically occur also in the northern part of Germany where the virus is carried by the striped field mouse (*Apodemus agrarius*). However, the causative agent responsible for human illness has not been previously isolated.

**Methodology/Principal Findings:**

Here we report on characterization of a novel cell culture isolate from Germany obtained from a lung tissue of “spillover” infected yellow necked mouse (*A. flavicollis*) trapped near the city of Greifswald. Phylogenetic analyses demonstrated close clustering of the new strain, designated Greifswald/Aa (GRW/Aa) with the nucleotide sequence obtained from a northern German HFRS patient. The virus was effectively blocked by specific antibodies directed against β3 integrins and Decay Accelerating Factor (DAF) indicating that the virus uses same receptors as the highly pathogenic Hantaan virus (HTNV). In addition, activation of selected innate immunity markers as interferon β and λ and antiviral protein MxA after viral infection of A549 cells was investigated and showed that the virus modulates the first-line antiviral response in a similar way as HTNV.

**Conclusions/Significance:**

In summary, our study reveals novel data on DOBV receptor usage and innate immunity induction in relationship to virus pathogenicity and underlines the potency of German DOBV strains to act as human pathogen.

## Introduction

Hantaviruses (*Bunyaviridae* family) are rodent-borne emerging viruses which cause two significant human diseases; hemorrhagic fever with renal syndrome (HFRS) in Asia and Europe, and hantavirus cardiopulmonary syndrome in the Americas. They carry a segmented, single-stranded RNA genome of negative polarity. There are several hantavirus species circulating in the world, each associated with a specific rodent or insectivorous host. The virus is transmitted to humans mainly through the inhalation of contaminated aerosols from rodent excreta and saliva [1].

In Europe Dobrava-Belgrade virus (DOBV) is the most life-threatening hantavirus leading to HFRS with case fatality rates of up to 12% [2], [3]. According to its natural hosts, mice of the genus *Apodemus*, DOBV forms distinct phylogenetic lineages [4]. DOBV-Af, represented by the original Dobrava isolate from Slovenia (Slo/Af), associated with *A. flavicollis* (Af) causes severe HFRS cases in the Balkan region. In *A. agrarius* (Aa) two lineages of hantavirus were found. DOBV-Aa, represented by the cell culture isolates SK/Aa from Slovakia and Lipetsk/Aa from Russia, is typical for Central Europe and Central European Russia [5], [6] where it causes mild/moderate disease. DOBV-like Saaremaa virus (SAAV), represented by cell culture isolate Saa/160V, from Estonia, North-East Europe [7], is not conclusively associated with clinical cases so far. Moderate to severe HFRS cases in South European Russia have been associated with DOBV-Ap lineage, represented by Sochi/Ap strain, transmitted by *A. ponticus* (Ap) [6]. Recently, a human isolate of DOBV-Ap, Sochi/hu, has been obtained from a fatal HFRS case [8].

In Germany, DOBV is endemic in the northern part of the country. Seroepidemiological studies including fine serotyping by neutralization assay as well as phylogenetic analysis of a patient-associated virus sequence showed that strains of the DOBV-Aa lineage are responsible for HFRS cases in this geographical region [5], [9]. Very recently, *A. agrarius* as reservoir host of DOBV-Aa lineage has been identified in three federal states of Germany. Moreover, multiple natural spillover infections (infections of rodent hosts other than the species identified as the predominant carrier for a particular virus) of *A. flavicollis* mice with DOBV-Aa have been reported [10]. Nevertheless, the causative agent of human disease from Germany was not isolated yet.

HFRS is characterized by symptoms as fever, microvascular hemorrhage, acute thrombocytopenia, hypotension, shock, and renal failure [11]. Hantavirus pathogenesis is believed to be a complex multifactorial process that includes β3 integrin dysfunction-mediated increase of vascular permeability, contributions of innate antiviral responses (cytokine storm), involvement of CD8^+^T-cells, and platelet dysfunction (reviewed in [11]–[14]).

Cellular β3 integrins are supposed to play an important role in hantavirus-mediated pathogenesis. They are primary regulators of endothelial cell barrier function and platelet activation [15], [16]. Usage of β3 versus β1 integrins as receptors for cell entry by hantaviruses seems to be one of the important pathogenicity determinants. So far it has been shown that β3 integrin is used by pathogenic Hantaan virus (HTNV), Seoul virus (SEOV), Puumala virus (PUUV), Sin Nombre virus (SNV), and New York-1 virus (NYV) while β1 integrin is utilized by non-pathogenic Prospect Hill virus (PHV) and low-pathogenic Tula virus (TULV) [15], [17], [18]. Recently it has been reported that HTNV utilizes also Decay Accelerating Factor (DAF; [19]) and/or glycoprotein gC1qR/p32 (also called p33 or HABP-1; [20]) during the cell entry process.

Differential cellular interferon (IFN) response to hantaviruses is also considered as one of the pathogenicity determinants. A readout marker for IFN bioactivity, which has been often used in characterization of hantaviruses, is the antiviral MxA protein [21]–[25]. The MxA protein belongs to the superfamily of dynamin-like GTPases and is involved in mediation of antiviral immune response against many viruses [26]. It has been shown that over-expression of MxA protein in cell culture can block hantavirus replication [27], [28]. MxA gene expression is tightly regulated by type I and III IFN [29]. Hantaviruses are weak inducers of type I (α/β) interferon, however, a recent study revealed that hantaviruses are able to induce type III IFN (λ1–3) in type I IFN-deficient Vero E6 cells which are routinely used for generation of hantavirus stocks [30]. Therefore, influence of Vero E6-derived type III IFN on earlier observed DOBV MxA induction patterns [25] has to be investigated.

Here we report on the isolation and characterization of a novel DOBV strain which can be taken as representative for hantavirus causing HFRS in northern part of Germany. Besides complete genetic characterization, we investigated biological properties of the virus which are currently considered as pathogenicity determinants such as receptor usage and induction of interferons.

## Results

### Virus isolation

During 2002–2008, 366 *Apodemus* mice were trapped in three federal states of Germany (Lower Saxony, Mecklenburg-Western Pomerania and Brandenburg). DOBV-IgG ELISA and RT-PCR revealed altogether 11 *A. agrarius* and *A. flavicollis* animals infected with viruses of the DOBV-Aa lineage [10]. Lung tissues from three of these animals (GER/08/118/Aa, GER/08/125/Aa, and a spillover infected rodent GER/08/131/Af) were used in the current study. Lung tissue suspensions were inoculated onto Vero E6 cells. After six weeks of blind passaging (three passages) DOBV infected cells were detected by immunofluorescence assay only for the GER/08/131/Af sample and the presence of viral RNA in the corresponding cell culture supernatant was confirmed by RT-PCR (data not shown). The new DOBV isolate was named Greifswald/Aa (GRW/Aa) according to the region where the infected animal had been trapped and its molecular clustering with the virus lineage DOBV-Aa (see below).

The fact that the cell culture isolation succeeded only from the spillover infected rodent raised the question of determinants of successful isolation procedure. Therefore, we retrospectively quantified virus load in the used tissue samples by quantitative real-time PCR (qPCR). Indeed, the highest virus load was observed in lungs of GER/08/131/Af mouse (290±94 copies per ng of total RNA) while GER/08/118/Aa and GER/08/125/Aa tissue samples were determined to contain 99±62 and 0.7±0.6 copies per ng of total RNA, respectively.

### Sequence analysis of the virus genome

Complete nucleotide sequences of all three virus genome segments were determined. The exact 5′ and 3′ termini of all three segments were inferred, too. The S segment was found to be 1,675 nucleotides (nt) long and its single open reading frame (ORF; nt position 36–1,325) encodes the viral nucleocapsid (N) protein of 429 amino acids (aa). The M segment is 3,644 nt long and encodes the glycoprotein precursor (GPC) of 1,135 aa (ORF position 41–3,448). The L segment is 6,532 nt long and encodes the viral RNA-dependent RNA polymerase of 2,151 aa (ORF position 38–6,493).

The sequence similarities between GRW/Aa and other DOBV isolates were in range from 79.8 to 89.3% on nucleotide and from 90.2 to 98.8% on amino acid level (Table 1). As expected, SK/Aa showed the highest sequence identity values with more than 96% aa sequence identity for all three segments. Interestingly, for the S segment sequences the lowest similarity values within the DOBV group were observed for SAAV/160V while for M- and L- segment sequences Sochi/hu showed the lowest similarity values. Since current ICTV species demarcation criterion lies on 7% for both N and GPC amino acid sequences, GRW/Aa clearly belongs to the DOBV species.

**Table 1:** Complete open reading frame nucleotide and amino acid sequence identities of GRW/Aa with other DOBV and HTNV isolates.

	% identity of GRW/Aa to other virus isolates					
	S-segment		M-segment		L-segment	
virus isolate	nt	aa	nt	aa	nt	aa
SK/Aa	89.3	98.8	86.6	96.3	86.7	97.3
Slo/Af	88.2	97.9	83.2	94.3	85.9	97.6
Sochi/hu	87.7	98.3	79.8	90.2	83.4	95.8
SAA/160V	87.4	97.4	87.0	95.9	88.0	97.2
HTNV/76-118	74.2	83.4	70.5	76.6	74.7	85.0

nt, nucleotides; aa, amino acids.

### Phylogenetic analyses

**Figure 1:**
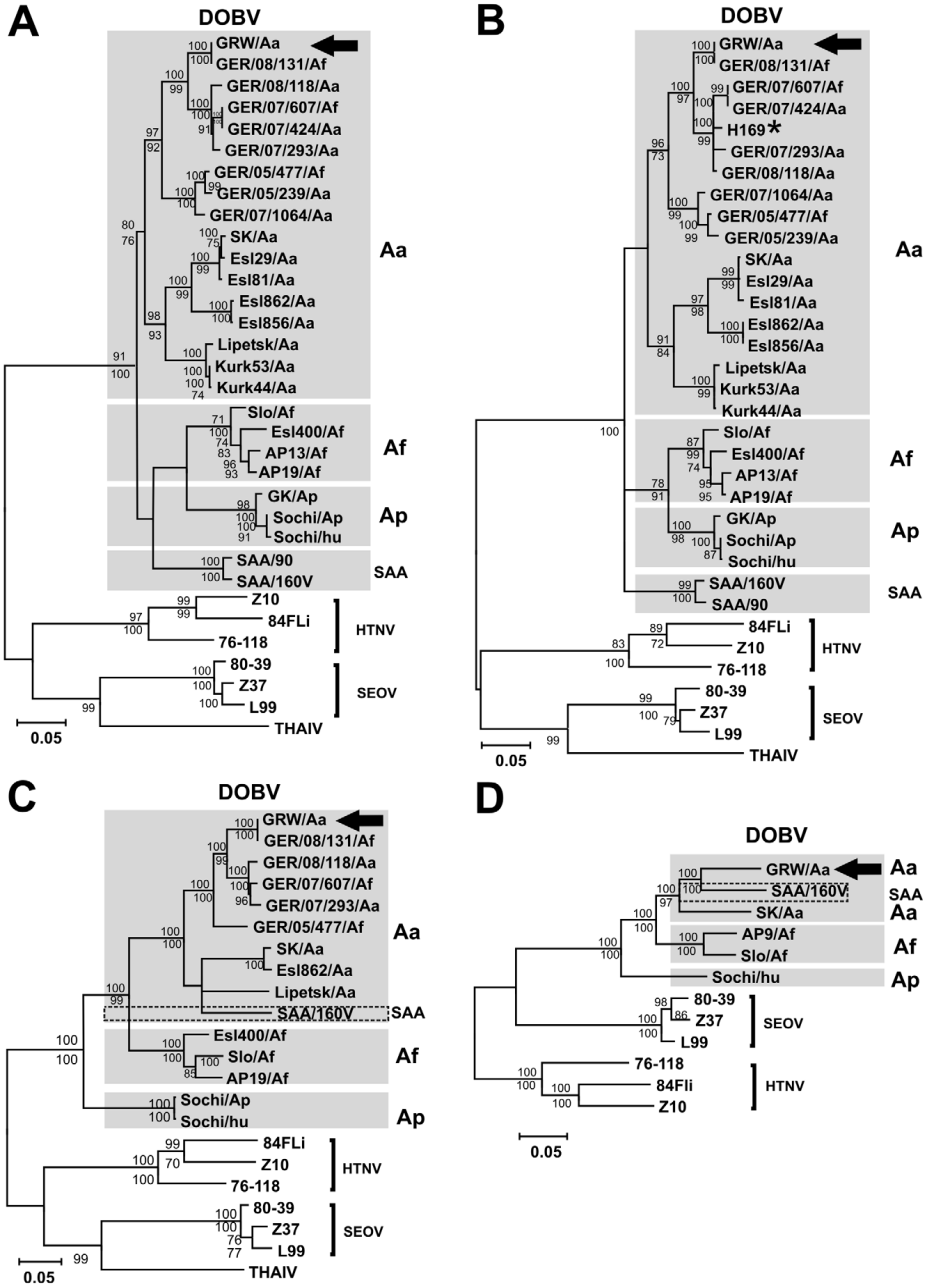
Maximum-likelihood phylogenetic trees of DOBV showing the phylogenetic placement of GRW/Aa. The trees were constructed with TREE-PUZZLE software (Tamura-Nei evolutionary model) and are based on (A) complete S-segment ORF, (B) partial S-segment (559 nt, positions 377 to 938), (C) complete M- and (D) complete L-segment ORF sequences. Values above the branches represent PUZZLE support values, while values below the branches are bootstrap values of the corresponding maximum likelihood trees calculated with the MEGA5 software from 1000 bootstrap pseudoreplicates. Only values >70% (considered significant) are shown. Different DOBV clades are indicated by gray boxes. GRW/Aa positions in the tree are designated with an arrow. H169 patient-derived sequence is designated with a star. For accession numbers, see the materials and methods. DOBV, Dobrava-Belgrade virus; HTNV, Hantaan virus; SEOV, Seoul virus; THAIV, Thailand virus.

Complete ORF sequences of all three GRW/Aa segments were analyzed by the Maximum Likelihood method (ML) with Tamura-Nei evolutionary model (Figure 1). Before the tree reconstruction all datasets were analyzed by the RDP3 program with automated recombination screening procedure [31]. No putative recombination regions could be detected by more than 3 programs implemented in RDP3. In all analyses, GRW/Aa clearly clustered within the DOBV species. Comparison of tree topologies from all three segments did not reveal any hints for genetic reassortment of the new virus isolate.

In the S and M segment analyses (Figure 1 A, C), the GRW/Aa sequences clustered with high statistical support within the DOBV-Aa evolutionary lineage. Analysis of the L segment ORF sequences is hampered by the fact that only very few complete sequences are available (Figure 1 D). As previously stated for the rodent tissue-derived sequence GER/08/131/Af [10], the new DOBV strain was isolated from *A. flavicollis* mouse, but genetically belongs to the DOBV-Aa lineage (Figure 1). This finding shows that the virus was obtained from a spillover infected animal. Therefore, the new isolate was designated as GRW/Aa according to its evolutionary origin in the DOBV-Aa lineage.

GRW/Aa formed a monophyletic group with sequences obtained from *Apodemus* mice trapped in northern Germany. No differences between the sequence obtained from the established cell culture isolate GRW/Aa and the GER/08/131/Af sequence obtained directly from the lung tissue were observed, demonstrating absence of mutations during the cell culture isolation procedure at least in the analyzed S and M segment coding sequences (Figure 1 A and C). Full length L segment ORF of GER/08/131/Af origin is not available.

Additional analysis based on partial S segment nucleotide sequences (559 nt) enabled inclusion of the only available DOBV-HFRS patient-derived sequence H169 [9] (Figure 1 B). Close clustering of GRW/Aa with H169 and mouse-derived DOBV-Aa sequences from northern Germany [10] demonstrated that GRW/Aa can be taken as a representative of pathogenic DOBV which is responsible for human HFRS cases in this geographical region.

### Cellular receptors necessary for GRW/Aa virus entry

**Figure 2:**
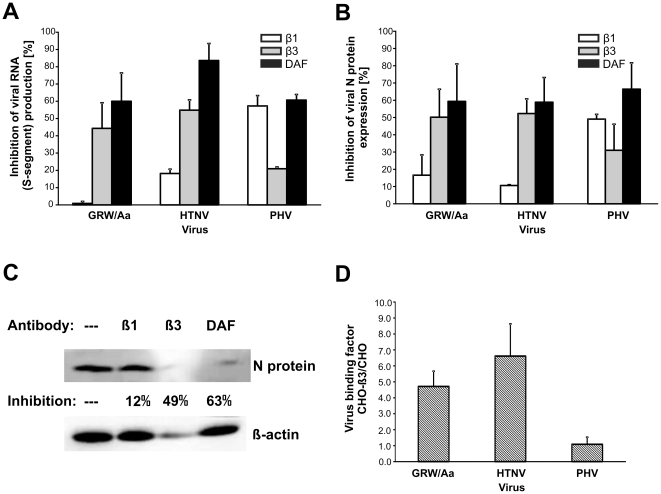
Determination of DOBV GRW/Aa receptor usage by receptor blocking assays (A–C) and receptor binding experiment (D). Vero E6 cells were treated with 40 µg/ml of indicated blocking antibodies for 1 hour. Then virus at multiplicity of infection 0.05 was added to the cells. After one hour cells were washed and new medium was added. One day later samples were collected. **A**) Viral S-segment RNA was measured by qPCR. **B**) Expression of viral nucleocapsid (N) protein was detected by Western blot. The density of bands on blots was quantified by ImageJ 1.41o programm (Wayne Rasband National Institutes of Health, USA). The percentages of antibody-mediated inhibition of viral infection were calculated in comparison to untreated but infected cells. Experiment was performed three times. Data are presented as the mean ± SD of the mean. **C**) Representative picture of the Western blot analyses summarized in part B. —, untreated cells; β1, cells pre-treated with β1 integrin specific monoclonal antibody (MAb); β3, cells pre-treated with β3 integrin specific MAb; DAF, cells pre-treated with DAF specific MAb. **D**) Binding of GRW/Aa to CHO cells stably expressing β3 integrins (CHO-β3cells) in comparison to control CHO cells. Virus binding was performed at 4°C for 1 hour. HTNV and PHV were used as controls. The amount of bound virus was measured through detection of viral RNA by specific qPCR. The binding affinity of virus particles to CHO-β3 cells is expressed as a ratio between virus genome equivalents detected on CHO-β3 cells and on the control CHO cells. Error bars represent standard deviations of the means from three experiments.

Receptor usage of the novel GRW/Aa virus was determined by blocking experiments. In the Vero E6 cell line the putative receptors (αvβ3 integrin, α5β1 integrin, DAF) were blocked by pre-incubation with specific blocking antibodies and then the cells were infected with the virus. Efficiency of the virus entry blockage was evaluated by quantification of viral RNA with qPCR (Figure 2 A) and of viral nucleocapsid protein by Western blot analyses (Figure 2 B, C) in the infected cells. In addition to GRW/Aa, HTNV and PHV were used in the assay as a control of our experimental settings because they were reported to be inhibited by β3 and β1 integrin specific antibodies, respectively [17].

Both experimental approaches revealed that the presence of accessible avß3 integrins and DAF molecules on the cell surface is important for GRW/Aa virus entry. We observed up to 60% inhibition of GRW/Aa infection in the presence of anti-αvβ3 integrin antibodies, whereas anti-α5β1 integrin antibody failed to block GRW/Aa infection. In addition, GRW/Aa infection could be efficiently blocked also by anti-DAF antibodies. HTNV and PHV were preferentially inhibited by anti-αvβ3 integrin and anti-α5β1 integrin antibodies, respectively, as previously reported. Moreover, both control viruses could be inhibited also by anti-DAF antibodies.

Since usage of β3 integrins is considered as important pathogenicity determinant, we performed an additional experiment to further confirm β3 integrin as a potential GRW/Aa entry receptor. Virus binding to β3 integrins was examined by comparison of virus binding to CHO cells (expressing no integrins on their surface) and CHO-β3 cells stably expressing β3 integrins (Figure 2 D). In this experimental setup, around 5-fold-higher level of GRW/Aa virus genome copies was detected on CHO-β3 cells than on CHO cells. In the same experiment, prototypical HTNV and PHV showed expected results; significantly higher amount of HTNV RNA was detected on CHO-β3 cells as compared with CHO cells while no significant difference in the amount of PHV RNA was observed (Figure 2 D).

### Induction of interferon in response to GRW/Aa infection

**Figure 3:**
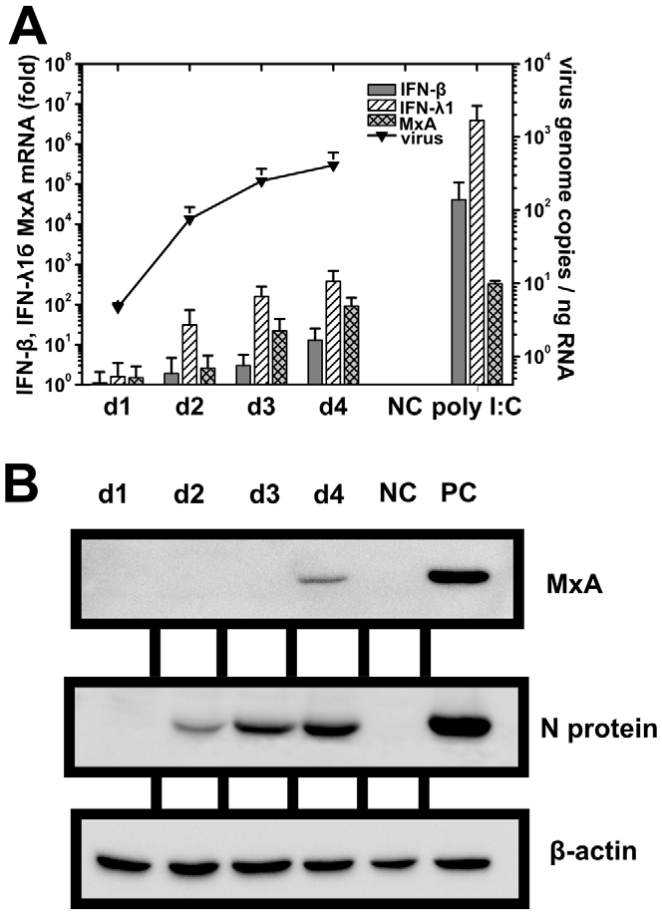
Expression of mRNA and antiviral MxA protein in response to GRW/Aa infection. A549 cells were infected at multiplicity of infection 1. Samples were taken at indicated time points post infection. **A**) Induction of IFN-β, IFN-λ1 and MxA mRNA (shown as fold of increase in comparison with uninfected cells) in response to GRW/Aa infection measured as virus genome copies by qPCR. Cells stimulated with poly I:C were transfected with 1.6 µg of poly I:C six hours prior infection. Experiment was performed three times. Data are presented as the mean ± SD of the mean. **B**) Expression of antiviral MxA protein detected by Western blot. NC (negative control), uninfected A549 cells. PC (positive control), A549 cells infected with HTNV for 4 days. Representative results of two independent experiments are shown.

First, induction of the interferon stimulated MxA protein expression in response to GRW/Aa infection in A549 cells was investigated on mRNA and protein level by qPCR and Western blot, respectively. MxA mRNA was detected from the first day and was gradually increasing from day 1 to day 4 post infection (Figure 3 A). However, MxA protein was first detected at day 4 post infection (Figure 3 B).

Since MxA expression is tightly regulated by both type I and type III interferon [29] induction of IFN-β and IFN-λ1 in A549 cells after GRW/Aa infection was further investigated by qPCR (Figure 3A). We observed delayed and weak induction of IFN-ß mRNA. On the other hand, induction of IFN-λ1 mRNA was considerably stronger, more rapid, and preceded the detectable induction of MxA and IFN-β genes.

**Figure 4:**
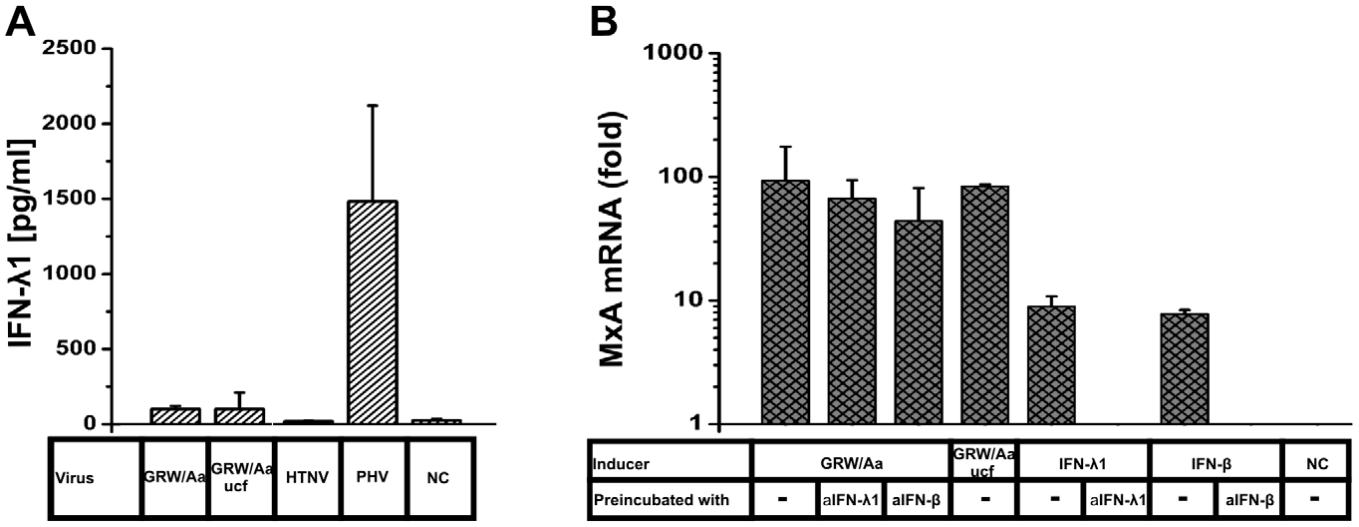
Influence of Vero E6-derived type III IFN on GRW/Aa MxA induction. **A**) 100 µl of indicated Vero E6-derived virus stocks or medium (NC, negative control) were exposed to UV irradiation. Amount of IFN-λ1 present in virus stocks was measured by ELISA. Abbreviation “ucf” means that the virus stock was purified by the ultracentrifugation procedure. Data are presented as the mean between three independently prepared stocks ± SD from the mean **B**) GRW/Aa virus stock (∼50 µl) or recombinant proteins (rhIFN-λ1 or rhIFN-β) were preincubated with 1 µg of corresponding blocking antibody (anti-IFN-λ1 or anti-IFN-β). One hour after incubation the mixture was added on the top of A549 cells. Four days post infection MxA mRNA expression was measured by qPCR. NC (negative control), untreated A549 cells. Experiment was performed three times. Data are presented as the mean ± SD of the mean.

Because it has been recently shown that hantaviruses may induce IFN-λ also in Vero E6 cells [30], we determined IFN-λ content in our Vero E6-derived GRW/Aa, HTNV, and PHV stocks in an IFN-λ1-specific ELISA. PHV and HTNV were used as controls because they were reported to elicit strong and weak IFN-λ secretion in Vero E6 cells, respectively [30]. The results revealed that GRW/Aa and HTNV stocks contained very low amounts of IFN-λ1 (∼100 pg/ml). In agreement with the previous report, PHV stocks contained substantially higher IFN-λ1 amount of up to 2,000 pg/ml (Figure 4 A).

Although the amount of IFN-λ detected in GRW/Aa stock was barely detectable, we wanted to distinguish whether the MxA induction observed after GRW/Aa infection of A549 cells (Figure 3) was caused just by passively transferred IFN-λ present in the virus stock or by *de novo* produced IFN induced by GRW/Aa virus infection (Figure 4B). For this purpose, we tried to eliminate residual IFN-λ activity in two ways. First, we purified the virus stock by ultracentrifugation and replaced the Vero E6 cell-derived supernatant by fresh cell culture medium (virus stock designated as GRW/Aa ucf in Figure 4). Second, we pre-incubated the virus stock with IFN-λ1- and IFN-β-blocking antibodies.

Clearly, neither ultracentrifugation nor pre-incubation of the GRW/Aa Vero E6-derived stock with anti-IFN-λ1 antibodies significantly inhibited MxA responses observed above. As a control for antibody specificity and efficiency, recombinant human IFN-λ1 and IFN-β along with anti-IFN-λ1 and anti-IFN-β neutralizing antibodies were used too. In summary, the obtained data indicate that the observed MxA induction pattern in A549 cells is due to GRW/Aa virus infection and not due to IFNs putatively present in the virus stock preparations.

## Discussion

DOBV-associated HFRS is endemic in northern Germany. Clinical cases of DOBV infected humans are reported regularly (Robert Koch-Institut: SurvStat, http://www3.rki.de/SurvStat) on the basis of serodiagnostics. However, as molecular proof of human infection by DOBV-Aa, only one short S segment sequence derived from a patient in North-East Germany has been obtained so far [9]. Recently, *A. agrarius* has been identified as the natural host of DOBV in this particular region [10]. Here we report on cell culture isolation and characterization of a DOBV strain from Germany which was designated as strain Greifswald (GRW/Aa). Close phylogenetic clustering of the isolate with the available sequence from the HFRS patient (Figure 2 B) and with additional German patient-derived sequences (J. Hofmann, personal communication) let us conclude that this virus is the causative agent of the human DOBV infections in the area.

Genome sequence and phylogenetic analyses clearly showed that GRW/Aa belongs to DOBV-Aa lineage (carried by *A. agrarius* as natural host), although it was obtained from an *A. flavicollis* mouse. This indicates that the virus was isolated from a spillover infected animal. To our knowledge, this is a first report on hantavirus cell culture isolation from a spillover infected animal. Interestingly, out of three isolation attempts, only the tissue from the spillover infected animal led to successful virus isolation. Tissues of spillover infected animals might be in fact better starting material for virus isolation attempts than tissues from the natural hosts. Spillover infections are assumed to be acute and transient and could therefore deliver higher virus loads than normally found in persistently infected natural hosts. Indeed, the virus load in the lungs of the spillover infected mouse (GER/08/131/Af) was found to be higher in comparison to virus loads in lungs of the other two *A. agrarius* mice (GER/08/118/Aa and GER/08/125/Aa).

Multiple DOBV spillover infections were recently observed in Germany [10]. Since hantaviruses have a three-segmented RNA genome, such spillover infections in nature can lead to the formation of a virus with a reassorted genome, as it has been observed for several hantaviruses [32]–[36]. Moreover, it has been recently shown that two strains, representing DOBV-Aa and DOBV-Af lineages, can be reassorted in cell culture [25]. However, our sequence and phylogenetic analyses of complete S-, M- and L-segments did not show any indications for reassortment and recombination events between GRW/Aa genome segments and other hantavirus strains (Table 1, Figure 1).

Hantavirus receptor usage is considered as one of the important pathogenicity determinants. Pathogenic hantaviruses were reported to bind inactive (bent) conformations of β3 integrin subunits, block endothelial cell migration and enhance the permeability of endothelial cells in response to vascular endothelial growth factor [11], [37], [38]. Although DOBV is the most virulent indigenous hantavirus in Europe, its receptor usage has not been determined yet. Therefore, we analyzed putative cellular receptors of GRW/Aa by antibody blocking experiments. Our data revealed that blocking of avβ 3 integrin and DAF molecules by specific antibodies inhibits GRW/Aa infection. We therefore conclude that these molecules are used as virus entry receptors. Obtained results extend the list of pathogenic hantaviruses reported to use β3 integrins as virus entry receptors. Moreover, not only GRW/Aa but also PHV as a “control virus” could be efficiently blocked by anti-DAF antibodies as previously shown for HTNV and PUUV [19].

Moreover, we investigated the induction of innate immunity markers in response to GRW/Aa infection. We observed delayed expression of antiviral MxA mRNA and protein after infection of A549 cells with GRW/Aa (Figure 3 A, B). On the protein level similar patterns were obtained for pathogenic HTNV and DOBV strain SK/Aa [24], [25]. It has been reported that pathogenic HTNV does not induce a strong type I IFN response in cell culture [21], [39], although MxA expression can be detected. Recently it has been shown that hantaviruses induce type III IFN in type I IFN-deficient Vero E6 cells [30]. Furthermore, Stoltz and Klingström reported that MxA is induced by IFN-λ1 (type III IFN) in a type I IFN-independent manner in A549 cells infected with HTNV [39]. We also detected only a minor induction of type I IFN on the mRNA level in A549 cells infected with GRW/Aa. However, consistent with published studies [30], [39], we found considerable induction of type III IFN in response to GRW/Aa infection (Figure 3 A). Occurrence of IFN-λ1 mRNA preceded the stimulation of MxA and IFN-β mRNA, suggesting that MxA expression is primary induced by type III IFN in GRW/Aa infected A549 cells.

In summary, our data demonstrate that the cell receptor usage and the MxA induction profile of GRW/Aa virus resemble those observed for HTNV. These findings underline the potency of GRW/Aa to act as a human pathogen.

## Materials and Methods

### Cells and viruses

Vero E6 (African green monkey epithelial kidney cell line; C1008 ATCC CRL 1586) and A549 cells (human epithelial lung cell line; ACC 107, German Collection of Microorganisms and Cell Cultures, Braunschweig, Germany) were cultured in Minimal essential medium (MEM) with Earle's Salt supplemented with 5% fetal calf serum, 25 mM HEPES, 1% glutamine, 1% sodiumpyruvate, 1% non essential amino acids, and 0.1% gentamycine sulphate. CHO-K1 cells stably transfected with pcDNA 3.1 vector (Invitrogen) expressing αV integrin and pZeoSV vector (Invitrogen, Germany) expressing β3 integrin (designated as CHO-β3 cells) as well as CHO-K1 cells transfected with empty control vectors (designated as CHO cells) were cultured and maintained in selection medium (50% HAMs F12, 50% DMEM, 10% FCS, 1% penicillin/streptomycin, 1% L-glutamine, 350 µg/ml G418 and 250 µg/ml Zeocin) [40].

Stocks of GRW/Aa, HTNV 76–118 (kindly provided by Dr. Åke Lundkvist), and PHV strain 3571 (kindly provided by Dr. Robert B. Tesh) were produced in Vero E6 cells cultivated in 75 cm^2^ cell culture flasks. The cells were infected at the multiplicity of infection (MOI) 0.1 for 1 hour at 37°C in 5% CO_2_ humidified atmosphere (standard cell culture conditions), then fresh medium was added. After seven days, cell culture supernatants were collected, centrifuged to remove cell debris, aliquoted and stored at −80°C. Virus stocks and cells were determined to be free of mycoplasma contamination by using the PCR-based VenorGeM mycoplasma detection kit (Minerva Biolabs, Germany).

### Virus ultracentrifugation

For production of high-titered, IFN-free virus stocks 175 cm^2^ cell culture flasks were infected and incubated for 7 days. After 2 freeze/thaw cycles, cells were scraped from culture vessel bottom and exposed to sonication. Cell debris was removed by centrifugation. Supernatant was transferred into sealed tubes and ultracentrifuged for 3 hours at 28,000 g and 4°C. Virus pellets were resolved in fresh culture medium through repetitive vortexing and sonication.

### Virus titration

Virus stocks were titrated with the chemiluminescence focus assay [41]. Briefly, confluent Vero E6 cells grown in 6-well plates were inoculated with tenfold dilutions of viral stock. After virus adsorption for 1 hour at culture conditions, the cells were overlaid with a 1∶1 mixture of 2.4% Avicel (FMC Biopolymer, USA) in water and basal Eagle's medium (2× BME). Plates were then incubated for 7–10 days (dependent on the virus strain) under the conditions described above. Virus-infected cells were detected with hantavirus N-protein specific polyclonal rabbit serum, followed by peroxidase-labeled goat anti-human IgG and chemiluminescence substrate Super Signal West Dura (Pierce, USA).

### Cell culture isolation procedure

DOBV reverse transcription-PCR (RT-PCR) positive lung samples from three naturally infected *Apodemus* mice (two *A. agrarius* and one *A. flavicollis*) trapped in a region near Greifswald (northern part of Germany [10]) were used for virus isolation attempts according to the previously described protocol [5]. In brief, mouse lung tissue suspensions (1 ml/flask) were inoculated onto cultures of confluent Vero E6 cells in 25 cm^2^ flasks. The cell culture medium was changed for the first time after 90 min and then weekly. In two-week intervals, cells were passaged into new culture flasks with the addition of the same amount of fresh uninfected cells according to a described protocol [7].

### Immunofluorescence assay (IFA)

To prove the success of virus isolation a standard IFA on 12-well spot slides with acetone-fixed cells was carried out as described previously [42]. Anti-DOBV human convalescent-phase serum was used to detect the virus.

### Reverse transcription-PCR (RT-PCR), cloning and sequencing

Total RNA from cells and mouse tissues was isolated with RNeasy Mini Kit (Qiagen, Germany) using the standard QIAamp viral RNA minispin protocol described by the manufacturer. Extracted RNA was directly reverse transcribed by use of SuperScript III First-Strand Synthesis System, following the application protocol of the manufacturer (Invitrogen, Germany). Initial screening, amplification and sequencing of the entire S- and M-segment sequences were performed as described previously for the DOBV SK/Aa isolate [5]. The primer sequences were designed from published DOBV entire S- and M-segment sequences and are available upon request. For the sequencing of the complete L-segment ORF, two long overlapping PCR fragments were prepared using primers binding to the L-segment termini MURL-F1 (5′-TAGTAGTAGACTCCSKAA-3′) and HTND-U1 (5′-TAGTAGTAGTATGCTCCGGAAA-3′) in combination with primers originally designed for the nested L-segment-based screening PCR [43]. The obtained long PCR products were then sequenced using the shotgun-based Long PCR Product Sequencing (LoPPS) protocol [44].

### Sequence and phylogenetic analysis

The obtained overlapping nucleic acid sequences were combined for analysis and edited with the aid of the SEQMAN program from the Lasergene software package (DNASTAR, Madison, Wis.). The sequence data were further analyzed by using the BioEdit software package [45]. Sequences were aligned by using CLUSTAL W, implemented in BioEdit software, with default parameters. The sequences were first aligned on amino acid level and then reverse translated to nucleotide sequences. The reliability of the alignment was checked by using DotPlot analysis provided by BioEdit software package. The alignment was tested for phylogenetic information by Likelihood Mapping analysis [46]. Before tree construction, automated screening for recombination between genomic segment sequences was performed using program RDP3 [31], which used 9 recombination detection programs: Bootscan, Chimeric, GENECONV, MaxChi, RDP, 3Seq, LARD, PhylPro and SiScan with their default parameters.

To construct maximum-likelihood (ML) phylogenetic trees, we applied quartet puzzling by using the TREE-PUZZLE package [46], [47]. As an evolutionary model for the reconstructions, the Tamura-Nei model was used; missing parameters were reconstructed from the datasets. In addition, ML phylogenetic trees (Tamura-Nei evolutionary model) were constructed also with the MEGA5 software [48] from 1,000 bootstrap pseudoreplicates. Resulting evolutionary trees were then visualized by using MEGA5 software [48]. All sequences obtained within this study have been submitted to the GenBank database under accession numbers JQ026204-6. Other nucleotide sequences used within the study were obtained from GenBank and their accession numbers are available upon request.

### Receptor blocking assay

Antibodies against α5β1 integrin (mouse monoclonal antibody anti-Integrin α5β1, clone JBS5, Millipore, Germany), αVβ3 integrin (mouse monoclonal antibody anti-Integrin αVβ3, clone LM609, Millipore, Germany), or DAF/CD55 (rabbit polyclonal antibody H319, Santa Cruz, Germany) were added to the confluent Vero E6 cells. Cells were treated with 40 µg/ml of antibodies for 1 hour at 4°C. Then the virus (MOI 0.05) was added to the monolayer. After incubation for 1 hour at 37°C, the cells were washed with medium and incubated for 24 hours under growth conditions. Samples were taken for qPCR and Western blot analysis. The percentages of antibody-mediated inhibition of infection were calculated in comparison to untreated, infected cells.

### Receptor binding experiments

CHO and stably transfected CHO cells stably expressing β3 integrins (CHO-β3 cells) were grown until confluence and cooled down to 4°C. Virus suspension (MOI of 0.5) kept at 4°C was added to the cells and incubated for 1 h in a refrigerator. After incubation, the cells were washed five times with cold medium, bound virus was lysed by RLT buffer from QIAamp RNeasy minikit (Qiagen, Germany) and used for RNA extraction according to the manufacturer's specifications. The binding affinity of virus particles to CHO-β3 cells was measured by qPCR as a ratio between the number of virus genome equivalents detected on CHO-β3 cells in comparison to those detected on CHO cells.

### Western blot analysis

For the MxA protein detection A549 cells (12-well plate, 90–95% confluent) were infected at an MOI 1 and protein extracts were harvested at days 1, 2, 3, and 4 after infection. For the receptor blocking assay Vero E6 cells were treated and samples were collected as described above. Protein extracts were separated in a 10% SDS-PAGE and blotted either onto PVDF membrane (MxA assay) or nitrocellulose membrane (hantavirus N protein assay, Whatman GmbH, Germany). Hantavirus N protein was detected with N protein-specific polyclonal rabbit serum [24] and β-actin with the mouse monoclonal antibody *ab6276* α (Abcam plc, Cambridge, UK). MxA was detected with the monoclonal antibody M143 (kindly provided by G. Kochs and O. Haller [49]). Signals were visualized by using Chemiluminescence super signal west dura kit according to the protocol supplied (Pierce, Perbio, Germany).

### Quantification of protein bands on Western blot

The density of N protein and β-actin (reference protein) bands on Western blot were quantified by ImageJ 1.41o program (Wayne Rasband National Institutes of Health, USA). The expression of N protein was normalised to the expression of β-actin. The percentages of antibody-mediated inhibition of viral infection (N protein expression) were calculated in comparison to untreated but infected cells.

### Monitoring of MxA, IFN-β, IFN-λ1 and viral RNA expression by quantitative real time PCR (qPCR)

A549 cells were seeded in 12-well plates at a density to achieve 90–95% confluence after overnight incubation at culture conditions. For poly I:C treatment, cells were transfected for 6 hours with 1.6 µg/well of high molecular weight poly (I:C) (InvivoGen, USA), and Lipofectamine 2000 (Invitrogen, Germany). Cells were infected with MOI 1. At indicated time points RNA was isolated using the RNAeasy kit (Qiagen, Germany). Extracted RNA was subjected to the DNase digestion (RNAeasy MinElute Cleanup Handbook 10/2010, Appendix C), following the protocol provided by manufacturer (Qiagen, Germany). Purified RNA was reverse transcribed by M-MLV Reverse transcription kit with the random hexamer primers (Invitrogen, Germany). MxA, IFN-β and IFN-λ1 relative mRNA expression was quantified by QuantiTest Sybr Green PCR kit (Qiagen, Germany). Each PCR reaction contained 4 µl of corresponding cDNA, 0.4 µM final concentration of each corresponding primer in a 20 µl of total reaction volume. PCR conditions were taken from manufacturer protocol; annealing temperature for MxA and IFN-β primers was 55°C, and 59°C for IFN- λ1 primer pairs. Using the Pfaffl method [50], data are presented as the fold change in gene expression normalized to an endogenous housekeeping gene porphobilinogen deaminase (PBGD) and relative to the untreated control. Annealing temperature and primers for PBGD qPCR were adapted from previously described protocol [51].

qPCRs for GRW/Aa, HTNV and PHV viral S-segments RNA were performed as previously described [52]. The viral RNA was quantified by using S-segment templates of known copy numbers. Virus genome copy numbers were normalized to ng of total cellular RNA. The list of primers and probes used for detection of gene and viral RNA expression is attached in supplementary data (Table S1).

### Inactivation of hantaviruses by UV irradiation

Virus stock solution (0.5 ml) was transferred to a small plastic petri dish and placed directly on the workspace of the UV transilluminator equipped with 8-W tubes (Vilber Lourmat, France). Inactivation was performed by UV irradiation for 3 min at 312 nm, corresponding to 1.4 J/cm^2^ [53].

### ELISA of IFN-λ1 and antibody blocking studies

Vero E6-derived virus stocks and Vero E6-conditioned medium (used as a negative control) were exposed to the UV irradiation (described above). The amount of IFN-λ1 was measured by ELISA using the human IL29 DuoSet ELISA Development System (R&D Systems, DY1598), following manufacturer instructions and modifications from recently described protocol [29].

For antibody blocking studies, virus or recombinant proteins were incubated with 1 µg/well of anti-IFN-λ1 or anti-IFN-β (R&D Systems, AF1598 and AF814, respectively) for 1 hour at room temperature. The resulting mixture was then added to A549 cells and incubated for 4 days prior the measurement of MxA mRNA expression. Recombinant proteins used included 2 ng/well of rhIFN-λ1 or rhIFN-β (R&D Systems, 1598-IL- 025 and 11415-1, respectively).

## Supporting Information

Table S1 Primers and probes used in real-time SYBR Green and TaqMan qPCR. *F = FAM label, **MGB** = Minor Groove Binder, **TMR** = TAMRA,
(DOC)
