# Supplementary material for: Dobrava-Belgrade Hantavirus from Germany Shows Receptor Usage and Innate Immunity Induction Consistent with the Pathogenicity of the Virus in Humans
Source: PLoS One. 2012 Apr 24;7(4):e35587. doi: 10.1371/journal.pone.0035587 (PMC3335829; doi:10.1371/journal.pone.0035587)
Supplement: Table S1 — Primers and probes used in real-time SYBR Green and TaqMan qPCR. *F = FAM label, MGB = Minor Groove Binder, TMR = TAMRA, (DOC) [file pone.0035587.s001.doc]

| Addressed gene | Primer/ Probe name | Primer/ Probe sequence* |
| --- | --- | --- |
| MxA | MxAq F | 5'-GAGGAGATCTTTCAGCACCTGAT-3' |
|  | MxAq R | 5'-CTGGATGATCAAAGGGATGTGGC-3' |
| IFN-β | IFNb se | 5'-GCCGCATTGACCATCTATGAGA-3' |
|  | IFNb as | 5'-GAGATCTTCAGTTTCGGAGGTAAC-3' |
| IFN-λ1 | IFN L1 F | 5'-GTCACCACAGGAGCTAGCGA-3' |
|  | IFN L1 R | 5'-GTGAAGGGGCTGGTCTAGG-3' |
| PBGD | PBGD F | 5'-ggCTgCAACggCggAA-3' |
|  | PBGD R | 5'-CCTgTggTggACATAgCAATgATT-3' |
| DOBV  S-segment | DOBV F | 5'-gACTCACCRTCATCAATYTgggT-3' |
|  | DOBV R | 5'-TggAggACAgMAAARAATgCACC-3' |
|  | DOBV R1 | 5'-gATgCCATgATIgTRTTCCTCAT -3' |
|  | Probe (DOBV MGB) | 5'-F-TCTgCCATgCCTgC--MGB-3' |
| HTNV  S-segment | Ht&Se F | 5'-CATggCWTCHAAgACWgTggg-3' |
|  | Ht&Se R | 5'-TTKCCCCATgCCACCAT-3' |
|  | Probe (Hat&Se MGB) | 5'-F-TCAATGGGGATACAACT--MGB-3' |
|  | Probe (Hat&Se MGB1) | 5'-F-TCAATGGGAATACAACT--MGB-3' |
| PHV  S-segment | lcPHVF | 5'-AGGAAGAGATCACTCGCCAT-3' |
|  | lcPHVR | 5'-TCCAATGTTGACACTGCTGA-3' |
|  | Probe (PHV-TAQ) | 5'-F-CATTGCCCGGCAGAAGCTCA--TMR-3' |
